# Supplementary material for: A Novel and Noninvasive Risk Assessment Score and Its Child-to-Adult Trajectories to Screen Subclinical Renal Damage in Middle Age
Source: Bioengineering (Basel). 2023 Feb 15;10(2):257. doi: 10.3390/bioengineering10020257 (PMC9952229; doi:10.3390/bioengineering10020257)
Supplement: Supplementary file 1 [file bioengineering-10-00257-s001.zip › bioengineering-2189393-supplementary.pdf]

bioengineering

MDPI

Supplementary Materials

A Novel and Noninvasive Risk Assessment Score and Its Child-to-Adult Trajectories to Screen Subclinical Renal Damage in Middle Age

Chen Chen [1,2], Guanzhi Liu [3], Chao Chu [1,2], Wenling Zheng [1,2], Qiong Ma [1,2], Yueyuan Liao [1,2], Yu Yan [1,2], Yue Sun [1,2], Dan Wang [1,2], Jianjun Mu [1,2,*]

[1] Department of Cardiovascular Medicine, First Affiliated Hospital of Xi'an Jiaotong University, Xi'an 710061 China
[2] Key Laboratory of Molecular Cardiology of Shaanxi Province, Xi'an 710061, China
[3] Department of Orthopedics, The First Affiliated Hospital, Zhejiang University School of Medicine, Hangzhou 310009, China
[*] Correspondence: mujjun@mail.xjtu.edu.cn

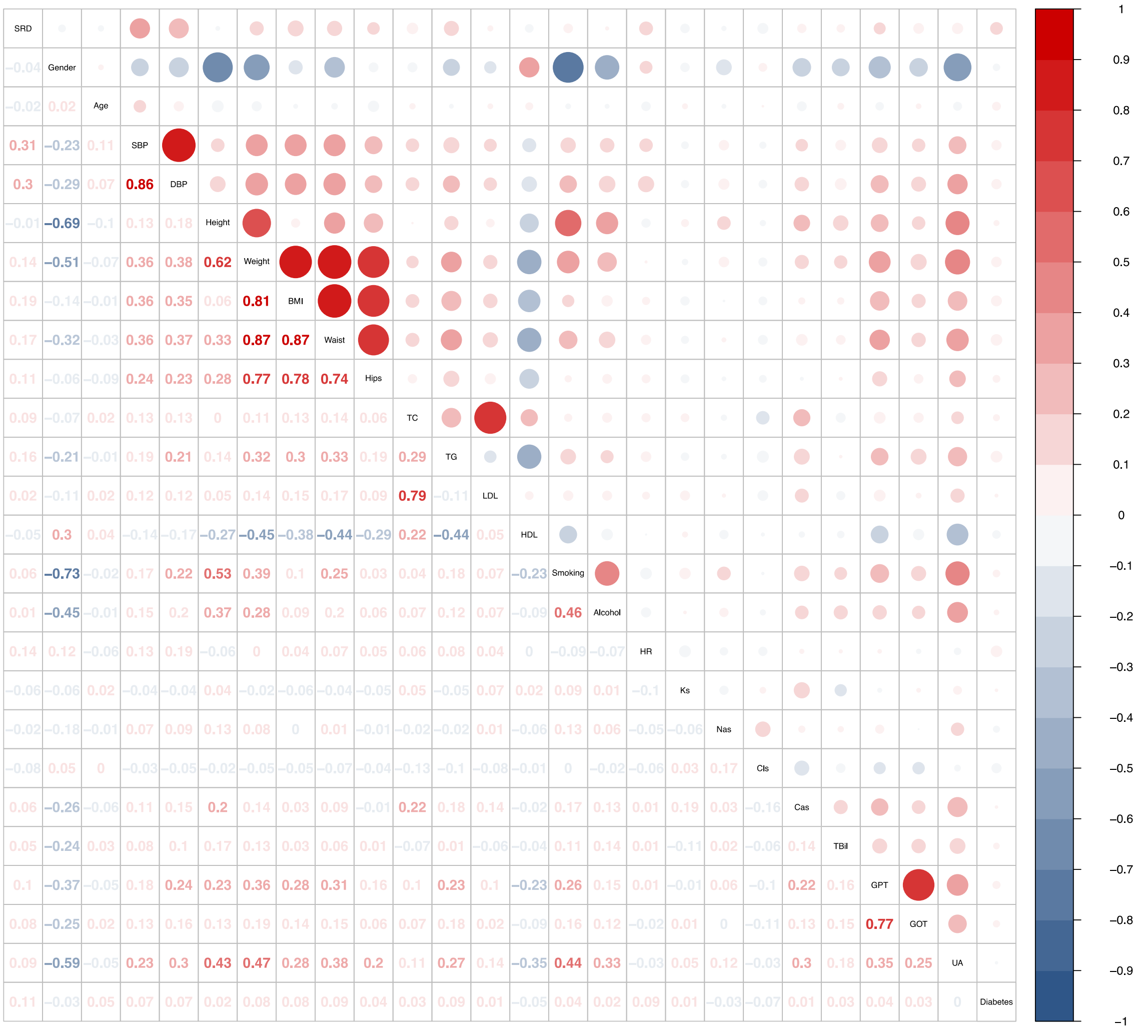

Figure S1. Heat map for the correlation between SRD-associated variables.
